# Dual mobility cups in primary total hip arthroplasties: trend over time in use, patient characteristics, and mid-term revision in 3,038 cases in the Dutch Arthroplasty Register (2007–2016)

**DOI:** 10.1080/17453674.2018.1542210

**Published:** 2018-11-19

**Authors:** Esther M Bloemheuvel, Liza N van Steenbergen, Bart A Swierstra

**Affiliations:** a Department of Orthopedic Surgery, Sint Maartenskliniek, Nijmegen;; b Dutch Arthroplasty Register (LROI), Hertogenbosch, the Netherlands

## Abstract

Background and purpose — We noticed an increased use of dual mobility cups (DMC) in primary total hip arthroplasty (THA) despite limited knowledge of implant longevity. Therefore, we determined the trend over time and mid-term cup revision rates of DMC compared with unipolar cups (UC) in primary THA.

Patients and methods — All primary THA registered in the Dutch Arthroplasty Register (LROI) during 2007–2016 were included (n = 215,953) and divided into 2 groups — DMC THA (n = 3,038) and UC THA (n = 212,915). Crude competing risk and multivariable Cox regression analyses were performed with cup revision for any reason as primary endpoint. Adjustments were made for sex, age, diagnosis at primary THA, previous operation, ASA score, type of fixation, surgical approach, and femoral head size.

Results — The proportion of primary DMC THA increased from 0.8% (n = 184) in 2010 to 2.6% (n = 740) in 2016. Patients who underwent DMC THA more often had a previous operation on the affected hip, a higher ASA score, and the diagnosis acute fracture or late posttraumatic status compared with the UC THA group. Overall 5-year cup revision rate was 1.5% (95% CI 1.0–2.3) for DMC and 1.4% (CI 1.3–1.4) for UC THA. Stratified analyses for patient characteristics showed no differences in cup revision rates between the 2 groups. Multivariable regression analyses showed no statistically significantly increased risk for revision for DMC THA (HR 0.9 [0.6–1.2]).

Interpretation — The use of primary DMC THA increased with differences in patient characteristics. The 5-year cup revision rates for DMC THA and UC THA were comparable.

The most frequent reason for revision in the 1st year after total hip arthroplasty (THA) is dislocation (LROI annual report 2016). Dislocation of a hip prosthesis is multifactorial including femoral head diameter. Mechanical studies have shown that instability could be decreased by increasing the diameter of the femoral head. With a larger head diameter, the head–neck ratio is higher and therefore there is a lower potential for instability (Burroughs et al. 2005).

Dual articulation implants were designed to increase implant stability but also to decrease polyethylene rim damage from contact between femoral neck and acetabular liner and to restore near-normal range of motion. The dual mobility cup (DMC) is a ‘cup in a cup’ and was developed in the 1970s to combine the low-friction arthroplasty principle of Charnley with the advantage of a big femoral head principle of McKee (Philippot et al. 2009).

Despite concerns about increased polyethylene wear due to the large femoral head, the DMC is not only used for revisions but also in primary THA to reduce dislocations. This was shown by De Martino et al. (2017) who counted, in a review of English articles between 1974 and 2016, 12,844 primary DMC THA and 5,064 revision DMC THA. Many of these articles focused more on dislocation rates than on longevity of the implant. Also in our daily practice we noticed an increase in the use of DMC in primary THA. Therefore, we determined the trend over time and mid-term cup revision rates of DMC compared with unipolar cup (UC) in primary THA with data from the Dutch Arthroplasty Register.

## Patients and methods

The Dutch Arthroplasty Register (LROI) is a nationwide population-based register that includes information on arthroplasties in the Netherlands since 2007. The LROI was initiated by the Netherlands Orthopaedic Association and is well supported by its members. This results in coverage of 100% of Dutch hospitals and a completeness of reporting of over 95% for primary THAs and 88% for hip revision arthroplasty (Van Steenbergen et al. 2015).

The LROI database contains information on patient, procedure, and prosthesis characteristics registered by registrars from each hospital. For each component a product number is registered to identify the characteristics of the prosthesis. The vital status of all patients is obtained actively on a regular basis from Vektis, the national insurance database on health care in the Netherlands, which records all deaths of Dutch citizens. The LROI uses the opt-out system to require informed consent of patients.

For the present study we included all patients that underwent a primary THA in the period 2007–2016. Metal-on-metal (MoM) THA (n = 6,626) and records with a missing product number (n = 7,017) were excluded. The remaining 215,953 hips comprised 3,038 DMC THAs and 212,915 UC THAs. Diagnosis was categorized as osteoarthritis (OA), acute fracture, late posttraumatic, and other. Other diagnoses registered in the LROI are dysplasia, inflammatory arthritis, osteonecrosis, post-Perthes, and tumor (unspecified). Cup revision was defined as a revision procedure where at least the cup was exchanged or removed. Closed reduction after a dislocation or incision and drainage for infection were not included in the LROI. The median follow-up was 3 years (0–9).

### Statistics

Survival time was calculated as the time from primary THA to 1st revision arthroplasty for any reason, death of the patient, or the end of the study follow-up (January 1, 2017). Cumulative crude incidence of revision was calculated using competing risk analysis, where death was considered to be a competing risk (Lacny et al. 2015, Wongworawat et al. 2015). In addition Kaplan–Meier survival analyses were performed.

Multivariable Cox proportional hazard ratios were performed to compare adjusted revision rates between DMC and UC THA. Adjustments were made for sex, age at surgery, diagnosis at primary THA, previous operation, ASA score, type of fixation, surgical approach, and diameter of the femoral head to discriminate independent risk factors for cup revision arthroplasty. BMI, Charnley score, and smoking status were not included as covariates, since these were only available in the LROI database since 2014. For all covariates added to the model, the proportional hazards assumption was met after inspecting log-minus-log curves.

Reasons for revision were described according to type of hip arthroplasty and compared using a chi-square test to test differences between types of THA (SPSS 22.0; IBM Corp, Armonk, NY, USA).

More than 1 reason could be chosen. P-values below 0.05 were considered statistically significant. For the 95% confidence intervals (CI), we assumed that the number of observed cases followed a Poisson distribution.

### Ethics, funding, and potential conflicts of interests

The dataset was processed in compliance with the regulations of the LROI governing research on registry data. No external funding was received. No competing interests were declared.

## Results

**Figure 1:**
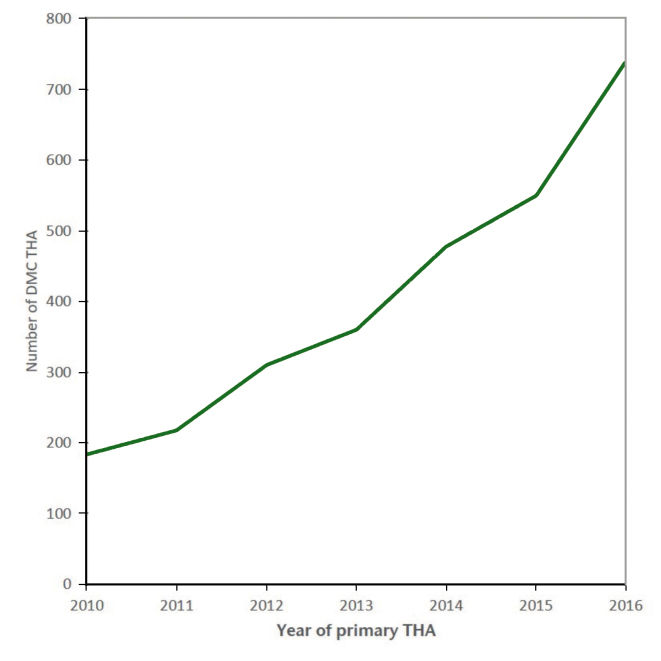
Trend in the use of dual mobility cup (DMC) in total hip arthroplasty (THA) in the period 2010–2016 in the Netherlands (n = 3,038).

The use of DMC THA increased from 184 (0.8% of all THAs) in 2010 to 740 (2.6% of all THAs) in 2016 (Figure 1) with 8 different types of DMC used (Table 1).

**Table 1.** Types of dual mobility cup THA used in the period 2007–2016 the Netherlands (n = 3,038)

Type	Cemented	Cementless
Biomet Avantage	1,904	84
Biomet Avantage Reload	–	339
Biomet Avantage Rev HA	–	5
Smith & Nephew Polarcup	79	273
Amplitude Saturne	164	85
Mathys SeleXys DS Cup	27	54
Groupe Lepine Cupule Quattro	17	–
Groupe Lepine Cupule HAP Press-F	–	7

In the DMC THA group more patients had undergone previous surgery on the affected hip and had a higher ASA score. Furthermore the distribution of diagnoses at primary surgery was different compared with the UC THA group (Table 2).

**Table 2.** Patient characteristics in THA according to type of acetabular cup (n = 212,915). Values are frequency and (%) unless otherwise specified

	DMC THA	UC THA
	n = 3,038	n = 212,915
Male sex, n (%)	1,104 (36)	70,144 (33)
Mean age (SD)	70 (13)	69 (11)
Operations before (yes)	632 (21)	10,048 (5)
ASA		
I	308 (10)	47,409 (22)
II	1,724 (57)	129,460 (61)
III–IV	951 (31)	27,748 (13)
Fixation		
Cemented	1,710 (56)	60,955 (29)
Hybrid (acetabulum cemented)	495 (16)	9,033 (4)
Hybrid (femur cemented)	126 (4)	9,932 (5)
Uncemented	674 (22)	130,911 (62)
Diagnosis		
Osteoarthritis	1,688 (56)	185,062 (87)
Fracture (acute)	424 (14)	7,065 (3)
Late posttraumatic	406 (13)	4,415 (2)
Other **^a^**	476 (16)	14,163 (7)
Approach		
Anterior	96 (3)	21,102 (10)
Anterolateral	41 (1)	15,801 (7)
Direct lateral	254 (8)	44,249 (21)
Posterolateral	2,607 (86)	128,275 (60)
Trochanter osteotomy	1 (0)	71 (0)
Other	8 (0)	635 (0)
Diameter (mm)		
22–28	2,784 (92)	66,703 (31)
32	–	93,619 (44)
36	–	4,002 (19)
≥ 38	–	1,452 (1)

Numbers do not add up to total due to missing data.

DMC: dual mobility cup; UC: unipolar cup; THA: total hip arthroplasty.

a Other: dysplasia, inflammatory arthritis, osteonecrosis, post-Perthes, tumor (unspecified).

**Figure 2:**
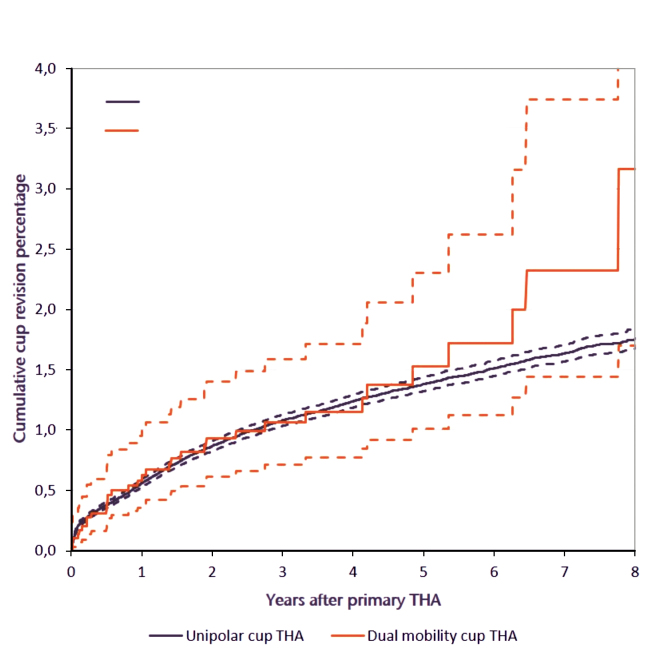
Cumulative incidence of cup revision according to type of cup (all diagnoses) in the period 2007–2016 in the Netherlands (n = 215,953). THA: total hip arthroplasty.

The 5-year crude cup revision rate for DMC THA was 1.5% (CI 1.0–2.3) and 1.4% (CI 1.3–1.4) for UC THA (Figure 2). Stratified analyses according to diagnosis at primary THA, previous surgery on the affected hip, and fixation of the cup showed similar 5-year crude cumulative incidence of cup revisions between the DMC and UC THA groups (Table 3 and 4, see Supplementary data).

The unadjusted hazard ratio for cup revision of DMC THA compared with UC THA was 1.2 (CI 0.8–1.6). Moreover, multivariable survival analyses showed a comparable risk for cup revision for DMC THA (HR 0.8 [CI 0.6–1.2]).

Dislocation was the most frequently registered reason for revision in UC THA patients (0.5%), while in the DMC THA group 0.2% were revised due to dislocation. In the DMC THA group loosening of the cup, dislocation, and infection were mostly registered as reason for revision (Table 5, see Supplementary data). From the 18 DMCs that loosened 8 were cemented.

## Discussion

We showed that the use of primary DMC THA increased in the Netherlands, with differences in patient characteristics between DMC and UC THA patients. The 5-year revision rates were comparable, with no differences in specific subgroups.

Our study is the first register study focusing on cup survival in primary use of DMC. Our 5-year cumulative incidences of cup revision of 1.5% in DMC THA and 1.4% in UC THA are lower than the overall revision rates from the Australian Orthopaedic Association National Joint Replacement Registry (2016), which reported 4.6% revision in 2,640 primary DMC THA and 3.3% in 327,847 primary UC THA. They did not specify the type of revision (insert, femoral head, cup, stem, or all). They also performed subgroup analysis and did not find a higher revision rate in any subgroup (AOANJRR 2016).

Our results differ from the Swedish Hip Arthroplasty Register (2016) where a hazard ratio of 2.4 for revision of Avantage DMC THA compared with UC THA after correction for case mix factors and after exclusion of infections was found. However, their result is also based on the overall revision rates, while our HR of 0.8 is based on cup revisions only.

Risk for cup revision due to dislocations was low with primary use of a DMC. In our study 8/3,038 (0.2%) DMC THA patients had a cup revision because of a dislocation versus 1,017/215,953 (0.5%) in UC THA. Tarasevicius et al. (2017) found in the Lithuanian Arthroplasty register at 5 years a revision rate for dislocation of 4/620 (0.7%) for primary DMC THA in comparison with 52/2,170 (2.4%) in a cemented Exeter cup. Revisions of UC THA are often preceded by 1 or more closed reductions (which are not reported in arthroplasty registers), while dislocations of DMC THA, being intra-prosthetic or not, are difficult to treat by closed reduction and will more often need surgery with exchange of components (which are reported in arthroplasty registers). So revision rates for dislocation in UC and DMC do not reflect instability in the same way.

(Suspicion of) infection was the second commonest reason for cup revision in the DMC THA group (10/36). In the LROI only (suspected) prosthetic joint infections as reason for revision were registered. As shown earlier, implant registries largely underscore prosthetic joint infections (Gundtoft et al. 2015) since incisions and drainages without component exchange are not included. In this respect Mukka et al. (2013) published a study of 34 hips with DMC THA with soft-tissue debridement of 3 hips due to superficial infection. Chughtai et al. (2016) reported 453 primary DMC THA with 2 septic revisions after 2 years. Differences in patient characteristics and particularly comorbidities are probably the explanation for our high amount (0.3%) of revisions due to suspected infection (Radtke et al. 2016). Furthermore, differences in hospital guidelines (early debridement in the case of wound problems), diagnosis, and treatment of implant infections could be a reason for more reported infections (Osmon et al. 2013).

Comparable risk for revision rates was seen between cemented and uncemented cups. Batailler et al. (2017) reviewed 21 studies with different cementless DMCs in primary THA with 0–8% aseptic loosenings after 2–22 years. They argued that the fixation of cementless DMC can be affected by poor bone quality. This could have been the case in patients with (post)traumatic diagnosis or other comorbidities.

Strengths of our study are, first, that the LROI contains a large population-based nationwide database of primary THAs, with a completeness of nearly 100% (van Steenbergen et al. 2015, LROI 2016) and an 8-year follow-up. Second, we focused our analyses on cup revisions, since type of revision (cup, stem, insert, and/or femoral head exchange) is specified in the LROI.

A limitation of this study is that in registries only limited variables are collected, correctness of data cannot be proven, and causality cannot be proven due to its observational nature. This might lead to residual confounding. Furthermore, closed dislocations are missed, since this procedure is not registered in the LROI, when no prosthesis component is added, exchanged, or removed. Dislocations for a DMC THA are almost always registered in the LROI since closed dislocation for DMC THA is most often impossible. Conversely, closed dislocation for UC THA can often be performed without surgery. This could lead to a lower revision rate in the UC THA group. The limited reliability of a diagnosis of infection has been discussed above.

In summary, the use of primary DMC THA in the Netherlands increased with differences in patient characteristics in comparison with UC THA. The 5-year revision rates for DMC THA were comparable to UC THA, even after adjustment for casemix factors. However, we need to be aware of residual confounding. To determine the exact role of DMC in primary THA compared with UC, randomized controlled trials or more subgroup analyses are needed.

### Supplementary data

Tables 3–5 are available as supplementary data in the online version of this article, http://dx.doi.org/10.1080/17453674.2018.1542210

All authors contributed to the conception of the study, data analysis, and preparation of the manuscript.

*Acta* thanks Ove Furnes and Per Kjaersgaard-Andersen for help with peer review of this study.

## Supplementary Material

Supplemental Material
