# Supplementary material for: Dual mobility cups in primary total hip arthroplasties: trend over time in use, patient characteristics, and mid-term revision in 3,038 cases in the Dutch Arthroplasty Register (2007–2016)
Source: Acta Orthop. 2018 Nov 19;90(1):11–4. doi: 10.1080/17453674.2018.1542210 (PMC6366470; doi:10.1080/17453674.2018.1542210)
Supplement: Supplemental Material [file IORT_A_1542210_SM5064.pdf]

Supplementary data

Table 3. Crude 5-year cumulative incidence (%) of cup revision according to type of cup

	DMC THA		UC THA	
	n	5-year cumulative incidence of revision (CI)	n	5-year cumulative incidence of revision (CI)
Overall (all records)	3,038	1.5 (1.0–2.3)	212,915	1.4 (1.3–1.4)
Diagnosis				
Osteoarthritis	1,688	1.6 (0.9–2.9)	185,062	1.3 (1.2–1.4)
Non-osteoarthritis	1,306	1.4 (0.8–2.4)	25,643	2.0 (1.9–2.2)
Previous surgery				
No	2,406	1.7 (1.1–2.7)	202,867	1.3 (1.3–1.4)
Yes	632	1.3 (0.5–3.2)	10,048	2.2 (1.9–2.5)
Cemented cup				
Yes	2,197	1.5 (0.9–2.6)	69,988	1.6 (1.5–1.7)
No	795	1.6 (0.8–3.1)	140,843	1.3 (1.2–1.3)

Numbers do not add up to total due to missing data
For abbreviations, see Table 2.

Table 4. Kaplan–Meier revision rates (%) according to type of cup

	DMC THA		UC THA	
	n	5-year cumulative revision rate (CI)	n	5-year cumulative revision rate (CI)
Overall	3,038	1.7 (1.0–2.5)	212,915	1.4 (1.4–1.4)
Diagnosis				
Osteoarthritis	1,688	1.7 (0.7–2.8)	185,062	1.3 (1.3–1.3)
Non-osteoarthritis	1,306	1.6 (0.8–2.4)	25,643	2.1 (1.9–3.0)
Previous surgery				
No	2,406	1.8 (0.9–2.9)	202,867	1.4 (1.4–1.4)
Yes	632	1.5 (0.1–1.6)	10,048	2.3 (2.0–2.7)
Cemented				
Yes	2,205	1.8 (0.6–3.0)	69,988	1.7 (1.5–2.7)
No	800	1.7 (0.7–2.7)	140,843	1.3 (1.3–1.3)

Numbers do not add up to total due to missing data.
For abbreviations, see Table 2.

Table 5. Reason for cup revision according to type of cup. Values are frequency and (%)

Reason for revision	DMC THA n = 3,038	UC THA n = 212,915
Dislocation	8 (0.2)	1,017 (0.5)
Infection	10 (0.3)	451 (0.2)
Periprosthetic fracture	3 (0.0)	98 (0.0)
Cup/liner wear	1 (0.0)	85 (0.0)
Girdlestone/spacer [a]	3 (0.0)	173 (0.1)
Loosening acetabular component	18 (0.5)	648 (0.3)
Loosening femoral component	2 (0.0)	227 (0.1)
Peri-articular ossification	1 (0.0)	40 (0.0)
Other	4 (0.3)	435 (0.2)

More than 1 reason could be listed as reason for revision.
[a] This reason for revision might be a result of registration error.
For abbreviations, see Table 2.
